# YOLOv10-LGDA: An Improved Algorithm for Defect Detection in Citrus Fruits Across Diverse Backgrounds

**DOI:** 10.3390/plants14131990

**Published:** 2025-06-29

**Authors:** Lun Wang, Rong Ye, Youqing Chen, Tong Li

**Affiliations:** 1 College of Mechanical and Electrical Engineering, Yunnan Agricultural University, Kunming 650201, China; 17300091752@163.com (L.W.); naniyafeng@163.com (Y.C.); 2 The Key Laboratory for Crop Production and Smart Agriculture of Yunnan Province, Yunnan Agricultural University, Kunming 650201, China; 15912913557@163.com; 3 School of Big Data, Yunnan Agricultural University, Kunming 650201, China

**Keywords:** YOLOv10, citrus defects, object detection, LDConv, precision crop protection

## Abstract

Citrus diseases can lead to surface defects on citrus fruits, adversely affecting their quality. This study aims to accurately identify citrus defects against varying backgrounds by focusing on four types of diseases: citrus black spot, citrus canker, citrus greening, and citrus melanose. We propose an improved YOLOv10-based disease detection method that replaces the traditional convolutional layers in the Backbone network with LDConv to enhance feature extraction capabilities. Additionally, we introduce the GFPN module to strengthen multi-scale information interaction through cross-scale feature fusion, thereby improving detection accuracy for small-target diseases. The incorporation of the DAT mechanism is designed to achieve higher efficiency and accuracy in handling complex visual tasks. Furthermore, we integrate the AFPN module to enhance the model’s detection capability for targets of varying scales. Lastly, we employ the Slide Loss function to adaptively adjust sample weights, focusing on hard-to-detect samples such as blurred features and subtle lesions in citrus disease images, effectively alleviating issues related to sample imbalance. The experimental results indicate that the enhanced model YOLOv10-LGDA achieves impressive performance metrics in citrus disease detection, with accuracy, recall, mAP@50, and mAP@50:95 rates of 98.7%, 95.9%, 97.7%, and 94%, respectively. These results represent improvements of 4.2%, 3.8%, 4.5%, and 2.4% compared to the original YOLOv10 model. Furthermore, when compared to various other object detection algorithms, YOLOv10-LGDA demonstrates superior recognition accuracy, facilitating precise identification of citrus diseases. This advancement provides substantial technical support for enhancing the quality of citrus fruit and ensuring the sustainable development of the industry.

## 1. Introduction

Citrus fruits have long maintained their position as the leading category in global fruit production and are also a vital economic crop in the southern regions of China [1]. The cultivation area encompasses 19 provinces located between 16° and 37° north latitude, including Guangxi, Hunan, Hubei, Guangdong, Sichuan, Jiangxi, Chongqing, Zhejiang, and Yunnan [2]. As a nationally recognized production area for extra-early- and extremely late-maturing citrus, Yunnan Province has witnessed the growth of its citrus industry into a significant pillar that drives economic development in the region.

The history of citrus cultivation in China dates back approximately 4000 years. Currently, China holds the position of the world’s largest producer of citrus, both in terms of cultivation area and production volume. However, the yield per unit area of citrus in China is significantly lower than the global average [3]. Diseases and pests are critical factors contributing to the decline in citrus yield and quality. In comparison to pests, citrus diseases exhibit more transmission routes and a broader spread, rendering them an ‘invisible killer’ that threatens the global citrus industry. Most citrus orchards in Yunnan are situated in mountainous regions, with individual orchards spanning hundreds or even thousands of acres. When a disease outbreak occurs, it can impact the entire supply chain of the production area. Therefore, timely detection and identification of diseases, along with targeted management strategies, are essential for minimizing losses in citrus orchards and ensuring both yield and quality.

With the iterative advancement of artificial intelligence technology, deep learning has transcended its traditional high-tech confines and has significantly permeated agricultural production. It is now applied in various scenarios, including pest and disease identification [4,5,6,7,8], maturity detection [9,10,11,12,13], and fruit grading [14,15]. Technologies based on deep learning have led to transformative breakthroughs in the agricultural sector. For instance, Shekhar, B. et al. [16] integrated YOLOv8 with drone imaging to propose a novel method for weed detection in soybean fields, thereby offering new insights into precision weeding. Li Daxiang et al. [17] designed a fine-grained knowledge distillation model to enhance the accuracy of lightweight convolutional neural networks in detecting apple leaf diseases and pests. Similarly, Zeng Weihui et al. [18] proposed a method for detecting citrus greening in natural backgrounds by integrating the Shearing Mixed Splicing (SMS) augmentation algorithm with bidirectional feature fusion, effectively addressing issues such as the missed detection of diseased leaves caused by the mutual occlusion of leaves in natural conditions. Additionally, Chen Y et al. [19] introduced an online citrus sorting system based on deep learning, capable of real-time detection of defective citrus through convolutional neural networks, thus achieving efficient citrus sorting. The YOLO series models are real-time object detection models based on deep learning that have been widely applied in the field of agricultural disease recognition due to their advantages, including real-time performance, high accuracy, and ease of deployment [20]. For instance, Sun Daozong et al. [21] enhanced the YOLOv4 model by introducing a convolutional attention module, which improved the recognition accuracy of tea white star disease, tea cloud leaf blight, and tea round spot disease. Additionally, Zhang, R. et al. [22] proposed the YOLO-CRD model, which improves upon YOLOv5s and increases the accuracy of detecting four types of rice diseases. Furthermore, Xie et al. [23] proposed the YOLO-EAF model based on YOLOv8n, which improved the detection accuracy of citrus greening disease in complex environments within citrus orchards.

Current research on citrus disease identification utilizing the YOLO series models reveals several shortcomings. On one hand, the detection accuracy of existing models does not fully satisfy the requirements for practical applications, particularly in complex environments characterized by variations in lighting, fruit occlusion, and subtle disease symptoms, which significantly impair model performance. On the other hand, the recognition rate for small lesions associated with citrus diseases remains low, resulting in frequent misdetections. In response to the aforementioned issues, this paper delves into optimizing detection methods for citrus diseases based on the YOLOv10 model. The LDConv module is employed to enhance the local feature extraction capability of the YOLOv10 Backbone network, effectively capturing the details of disease spots. The GFPN module is introduced in the Neck, integrating cross-scale feature fusion with a global attention mechanism to enhance the multi-scale representation of small target lesions. The DAT mechanism emphasizes the identification of critical discriminative regions within cross-scale features by employing a spatial-channel collaborative attention approach. This methodology effectively mitigates the discrepancies associated with similar diseases. Additionally, the AFPN is incorporated to improve the detection Head, suppressing background interference and enhancing adaptability to complex scales. The Slide Loss function is employed to dynamically weight challenging samples based on the Intersection over Union (IoU) metric. This approach alleviates class imbalance issues and effectively addresses the low recognition rate of small citrus disease spots, as well as the problem of disease misdetection across varying backgrounds.

This study is grounded in the YOLOv10 model, systematically integrating the LDConv, GFPN, DAT, and AFPN modules, along with Slide Loss, to develop the YOLOv10-LGDA model. This model achieves precise multi-scale lesion localization and effectively discriminates between similar diseases in complex orchard scenarios, thereby offering enhanced visual technical support for the advancement of the citrus industry.

## 2. Materials and Methods

### 2.1. Citrus Fruit Disease Dataset

**Figure 1:**
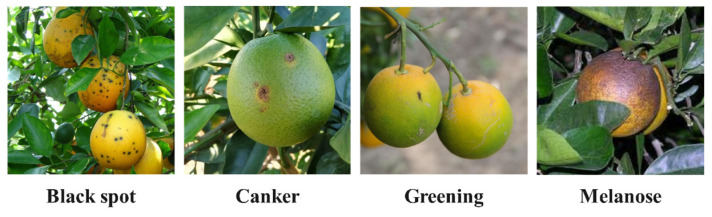
Images of different citrus fruit diseases.

This study focuses on four significant citrus fruit diseases: citrus black spot, citrus canker, citrus greening and citrus melanose. The characteristics of these four diseases are illustrated in Figure 1.

Citrus black spot primarily infects the epidermal tissue of the fruit, leading to a reduction in fruit yield [24,25]. Initially, small light brown spots appear on the fruit surface, which later expand into circular or irregular black spots. The center of the lesions is sunken and densely covered with small black dots. In severe cases, the lesions merge, causing the fruit surface to become rough. Citrus canker affects the fruit, branches, and leaves of citrus plants [26]. In the early stages of fruit infection, pinhead-sized yellow oily spots are visible. These spots then become raised, forming grayish-white crater-like cracks surrounded by a yellow halo. The skin around the lesions becomes uneven due to suberization, often presenting as blister-like lesions, which significantly reduces the fruit’s value [27]. Citrus greening induces the typical “red nose fruit” phenotype, characterized by abnormal orange-red coloration at the pedicel end, while the distal part remains green, along with small fruit size, uneven surface coloration, and fruit deformity [28]. Citrus melanose primarily affects citrus leaves, branches, and fruits, inhibiting the accumulation and transport of photosynthetic products. This results in restricted new shoot growth and abnormal fruit development, ultimately leading to tree decline and reduced yield. Typical symptoms during storage include the initial appearance of light brown, water-soaked patches on the fruit surface, which subsequently expand to form dark green, velvety mold layers that thicken under humid conditions, eventually causing fruit softening and decay.

The citrus fruit disease image dataset utilized in this study was predominantly sourced from the Kaggle open website, with a smaller portion collected from the Chu Orange Base in Longling County, Baoshan City, Yunnan Province, China (24.25 N, 99.06 E). The dataset comprises 1270 images of citrus fruits, categorized into healthy and four common pathological states. The image backgrounds encompass both complex natural environments and simple artificial settings, with the natural backgrounds presenting challenges such as leaf and branch obstructions, overlapping fruits, strong light reflections, and backlighting. All images maintain a uniform resolution of 640 × 640 pixels. The dataset comprises 268 images of healthy fruits, 249 images exhibiting citrus black spot, 233 images depicting citrus canker, 371 images associated with citrus huanglongbing, and 149 images illustrating citrus melanose. The samples of black spot display a continuous variation in lesion density, ranging from sparse to dense, while the canker samples encompass typical lesion features of varying sizes and morphologies. The greening samples present fruit phenotypes with differing degrees of deformity, and the melanose samples primarily showcase two typical phenotypes: water-soaked patches and velvety mold layers. The former is characterized by alterations in the light transmittance of the peel, while the latter is distinguished by the three-dimensional stacking of mycelial masses. By constructing a sample set that incorporates typical morphological differences and key lesion stages, the deep learning model’s capacity to recognize the spatial distribution characteristics of lesions can be enhanced, thereby improving the identification of small-sized lesions and early-stage lesions in various complex field environments.

To improve the accuracy of recognition and the generalization capability of the enhanced YOLOv10-LGDA model for different citrus fruit diseases in various environments, data augmentation strategies were utilized on the dataset images. These strategies featured horizontal and vertical rotations, along with adjustments in brightness, leading to the generation of a total of 7620 augmented images. The images were labeled utilizing the LabelImg tool and then partitioned into training, validation, and testing sets with a distribution of 8:1:1 for the experiments.

### 2.2. Improved YOLOv10 Overall Structure

#### 2.2.1. YOLOv10

YOLOv10 [29,30] proposes a multi-level collaborative optimization framework that overcomes the computational representation equilibrium bottleneck of traditional models through dynamic topology networks. This framework employs a gradient-guided parameter reorganization strategy to achieve joint optimization of feature extraction and inference efficiency. Its dual-stage supervision mechanism enhances representation learning by utilizing probability density label assignment during training and replaces non-maximum suppression with differentiable mapping during inference, effectively balancing detection accuracy and computational cost. By integrating spatial channel interaction attention with feature distillation techniques, the model concurrently optimizes inference efficiency, localization accuracy, and generalization ability through input-aware dynamic computation paths, thereby establishing an efficient perception paradigm for real-time vision systems.

YOLOv10 adopts a hierarchical feature processing architecture, which includes a feature extraction Backbone network (Backbone), a feature fusion Neck (Neck), and a dual-modal prediction Head (Head). Together, these components form a collaboratively optimized detection framework. The Backbone section dynamically optimizes network layers using differentiable architecture search technology, specifically designing lightweight C2f units tailored for real-time detection scenarios. It replaces traditional fixed structures with dynamic depthwise separable convolution kernels, achieving an optimal balance between parameter efficiency and receptive field preservation. The core innovation lies in the integration of the Position Self-Attention (PSA) module, which enhances key features through a coupling mechanism that combines spatial position encoding and channel attention. The Neck section is designed with a bidirectional heterogeneous feature pyramid that employs adaptive gating coefficients to regulate the interaction of top-down and bottom-up cross-scale information, effectively addressing the semantic gap issue in multi-level feature fusion. The Head section innovatively proposes a dual-stage collaborative training paradigm: during the training phase, a one-to-many Head is utilized to implement dense supervision, enhancing the model’s representational capability through a probability-driven label assignment strategy. During the inference phase, it switches to a one-to-one prediction branch, achieving a deterministic mapping of prediction results based on differentiable optimal transport theory. By designing a feature space alignment constraint mechanism, this architecture maintains the advantages of end-to-end training while fundamentally improving inference efficiency.

**Figure 2:**
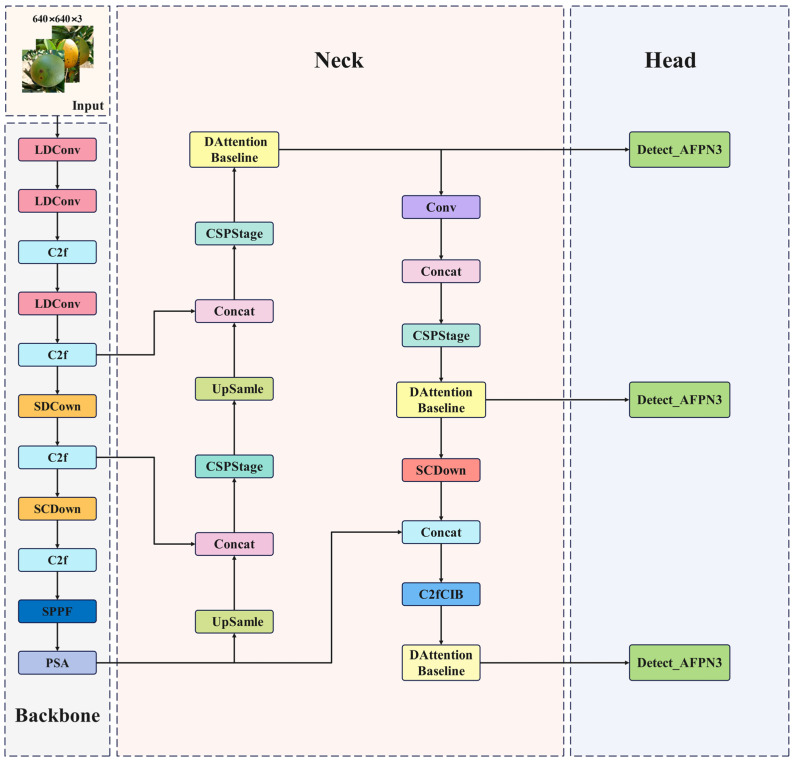
Improved YOLOv10n network structure diagram.

Deep learning-based detection methods demonstrate significant advantages in recognizing plant diseases within complex agricultural environments, thereby enhancing the robustness of disease identification under intricate conditions [31]. Multi-level feature extraction and dynamic attention mechanisms effectively address challenges such as variations in field lighting, foliage occlusion, and background interference. This paper introduces relevant improvements based on YOLOv10n to achieve efficient detection of citrus defects in these complex scenarios. The improved structure is illustrated in Figure 2.

#### 2.2.2. LDConv Module

Currently, convolutional neural network (CNN) architectures, which leverage characteristics such as local perception and weight sharing, have become the core paradigm for computer vision tasks [32,33]. However, their reliance on fixed-size convolution kernels and rigid grid sampling mechanisms makes it challenging to adapt to target features with irregular geometric shapes, such as lesions. The fixed and limited receptive field of conventional convolution processes can result in the gradual loss of features from early-stage small lesions due to downsampling operations as they propagate through deep networks. Deformable convolution enhances the flexibility of feature extraction by introducing offset parameters that dynamically adjust sampling positions. However, the parameter scale of this mechanism grows quadratically with the size of the convolution kernel, leading to a significant increase in computational complexity when large-sized convolution kernels are employed.

**Figure 3:**
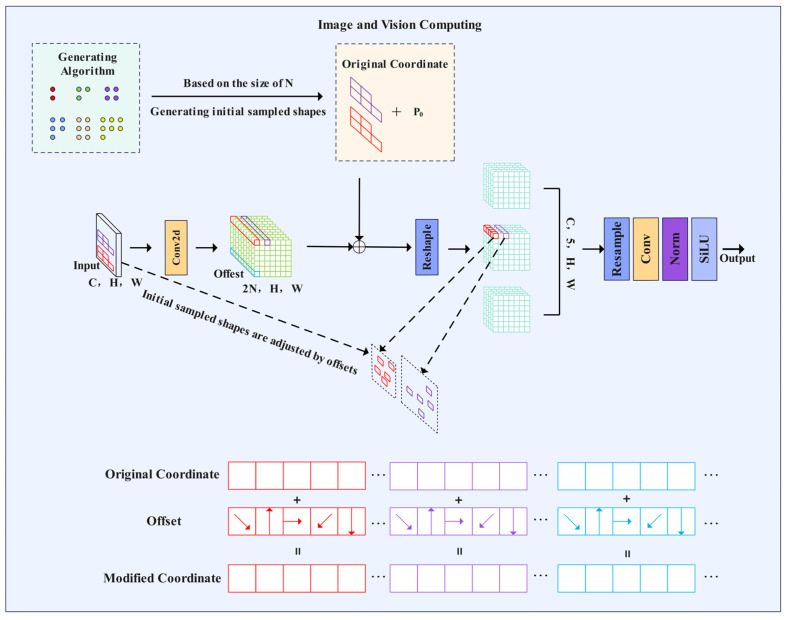
LDConv network structure diagram.

The innovative design of the LDConv (Learnable Deformable Convolution) [34] module effectively addresses the aforementioned issues. This module comprises three key components: a dynamic sampling coordinate generator, a learnable offset prediction network, and a multi-scale feature aggregation unit. During the dynamic sampling phase, LDConv generates a set of base sampling coordinates that are no longer confined to a regular grid distribution; instead, they obtain an initial sampling pattern through adaptive learning based on the input features. The offset prediction network predicts two-dimensional offsets for each sampling point, enabling the sampling locations to align precisely with the key feature regions of the lesion. Depthwise separable convolutions are employed to construct the offset prediction network, ensuring that the number of parameters increases only linearly with the size of the convolutional kernel, which significantly reduces computational complexity. The LDConv network structure is illustrated in Figure 3, where it assigns initial sampling coordinates to convolutions of arbitrary sizes and adjusts the sample shape using learnable offsets, thereby altering the sampling shape at each position through resampling.

In the feature aggregation stage, LDConv introduces a multi-scale fusion strategy that combines sampled features from various initial scales through weighted aggregation. This approach enables the module to simultaneously capture local details and global contextual information of lesion areas. When integrated with YOLOv10, a progressive replacement strategy is employed: small-sized LDConv (3 × 3) is utilized in shallow network layers to capture subtle lesion features, while large-sized LDConv (7 × 7) is applied in deeper network layers to model global contextual relationships. This hierarchical design facilitates a balanced trade-off between computational efficiency and effective feature extraction.

#### 2.2.3. Neck Network Improvements

**Figure 4:**
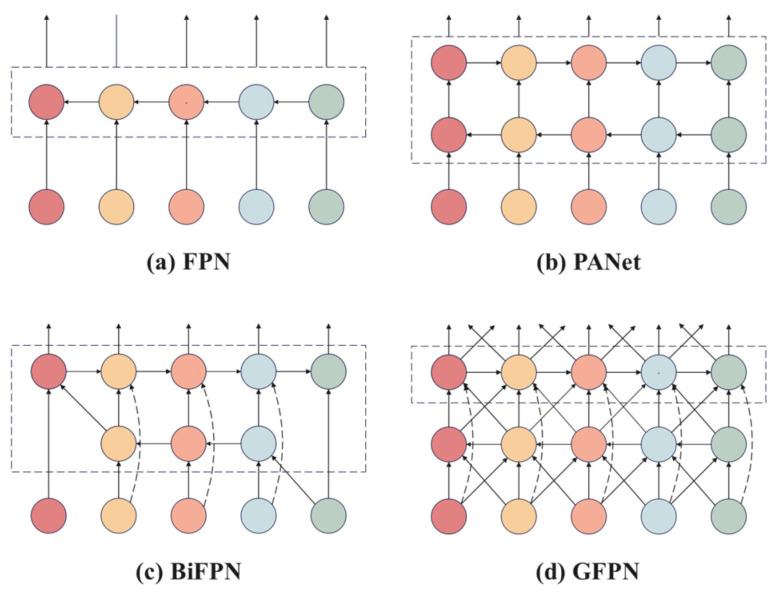
Comparison of four different Feature Pyramid Network structures.

Multi-scale feature fusion is a critical component in the design of object detection network architectures. Traditional Feature Pyramid Networks (FPNs) [35,36] transmit high-level semantic features through a top-down unidirectional fusion mechanism; however, this approach is prone to gradient attenuation during backpropagation and fails to fully integrate low-level spatial detail information. Although the Path Aggregation Network (PANet) [37] introduces a bottom-up auxiliary path to enhance feature representation, its dual-path structure significantly increases computational complexity. The Bi-directional Feature Pyramid Network (BiFPN) [38] improves computational efficiency by streamlining node connections; however, cross-scale interactions between feature layers remain insufficient. Furthermore, the weight allocation strategy does not adequately consider the complementary differences between fine-grained lesion textures in shallow high-resolution features and deep semantic features, which limits the effective integration of multi-level information. To address the aforementioned challenges, this study introduces the Global Feature Pyramid Network (GFPN), a module that facilitates the dynamic fusion of multi-level features through the establishment of a bidirectional interaction mechanism. The GFPN creates cross-level feature interaction channels by implementing skip-layer connections, which not only preserve the integrity of gradient propagation but also allow for the deep fusion of features across different abstraction levels. Additionally, it incorporates a cross-scale connection mechanism that dynamically aggregates features from adjacent layers via a learnable weight allocation strategy, thereby effectively balancing the representation of high-level semantic information and low-level spatial details. Experimental results demonstrate that this architecture enhances feature representation capabilities in complex scale-variant scenarios, significantly improving detection accuracy while maintaining computational efficiency, particularly excelling in challenging tasks such as small object detection and occluded object recognition. Different FPN architectures are illustrated in Figure 4, where Figure 4a–d depict the network structures of FPN, PANet, BiFPN, and GFPN, respectively.

The GFPN incorporates both skip-layer connections and cross-scale connections. The purpose of the skip-layer connections is to alleviate the vanishing gradient problem that may arise during backpropagation within the intricate Neck structure of the “Giraffe” [39]. Consequently, two feature connection methods are proposed: dense-link and $\log_{2}n-link$. In the dense-link approach, for each scale feature $P_{k}^{l}$ at level k, the *l*-th layer receives feature maps from all preceding layers, as illustrated in Equation (1).

(1) $$P_{k}^{l}=Conv(Concat(P_{k}^{0},\ldots ,P_{k}^{l-1})),$$

The $\log_{2}n-link$ mechanism is designed such that at each hierarchical level k, the L-th layer aggregates feature maps from up to $\log_{2}l+1$ preceding layers, where these input layers are exponentially spaced with base 2 in depth (*i*) as formulated in Equation (2).

(2) $$P_{k}^{l}=Conv(Concat(P_{k}^{l-2^{n}},\ldots ,P_{k}^{l-2^{1}},P_{k}^{l-2^{0}})),$$

The proposed $\log_{2}n-link$ architecture satisfies the condition ${l-2}^{n}\geq 0$ and achieves a time complexity of $O(l\cdot \log_{2}l)$ at depth *l*, significantly lower than the $O(l^{2})$ complexity of dense-link counterparts. Furthermore, it mitigates gradient degradation by maintaining shorter inter-layer propagation distances during backpropagation, thereby enhancing scalability for deeper neural architectures.

**Figure 5:**
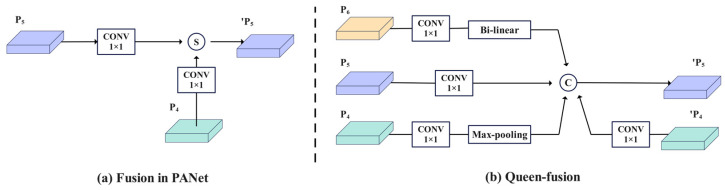
Schematic comparison of cross-scale feature fusion between PANet and GFPN.

Cross-scale connection aims to address the challenges posed by significant variations in target scale through multi-level feature interactions. Its essence not only depends on skip-layer connections but also necessitates a deep integration of cross-resolution features. The cross-scale fusion method, referred to as Queen-fusion, is introduced to enhance the integration of features across the same and adjacent levels. The Queen-fusion connection at layer P_5_ incorporates downsampling from the previous layer P_4_, upsampling from the subsequent layer P_6_, and features from both the current layer P_5_ and the previous layer P_4_. In practice, bilinear interpolation is utilized for upsampling, while max pooling is employed for downsampling. Here, P_4_, P_5_, and P_6_ represent feature maps at varying levels within the feature pyramid. By employing the Queen-fusion cross-scale connection method, features from different levels and layers are effectively integrated, facilitating a comprehensive exchange of high-level semantic information and low-level spatial information, thereby enhancing detection performance in large-scale, variable scenes. The feature fusion architecture is illustrated in Figure 5, where Figure 5a presents the PANet feature fusion diagram, and Figure 5b shows the GFPN feature fusion diagram. In this context, S and C denote the summation and concatenation fusion methods, respectively, while $P_{K}$ signifies the subsequent layer node.

#### 2.2.4. Deformable Attention Module

**Figure 6:**
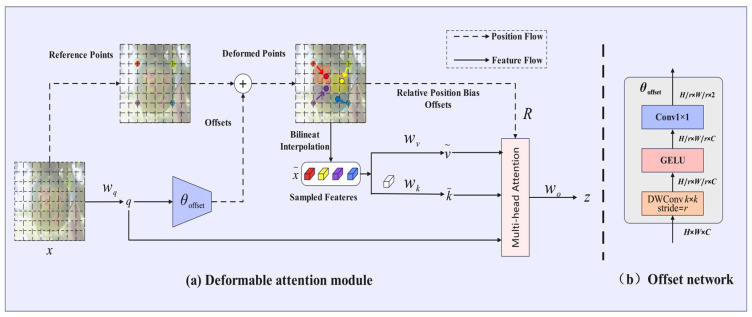
DAT network structure diagram.

Citrus fruits frequently thrive in dense foliage environments, where mutual occlusion between fruits, as well as between fruits and branches, is prevalent. This phenomenon complicates the ability of traditional detection algorithms to accurately identify partially occluded diseased areas. Additionally, the morphological characteristics of citrus diseases exhibit significant changes throughout the various stages of disease development, transitioning from initial spot-like lesions to later ulcer-like lesions. These lesions differ in shape and possess irregular boundaries, presenting a substantial challenge to traditional convolutional neural networks that rely on fixed receptive fields. The Deformable Attention (DAT) [40] module transcends the rigid geometric constraints of traditional attention mechanisms by learning dynamic offset vectors. Its essence lies in the construction of a differentiable offset field, which allows the attention window to adaptively deform based on the semantic features of the target. For the input feature map, the DAT generates per-pixel spatial offset vectors through a lightweight offset prediction network, guiding the attention weights to dynamically concentrate on the effective semantic regions of the target. In occlusion scenarios, this mechanism automatically adjusts the distribution of sampling points via offset vectors, suppressing interference signals from occluded areas while enhancing the response intensity of visible pathological features. For targets with irregular shapes, the DAT employs a multi-scale offset compensation strategy, enabling attention sampling points to adaptively distribute along the pathological topological structures, thereby achieving pixel-level alignment between lesion geometry and the attention field. This characteristic of semantic-driven deformation allows the model to perceive local details of complex targets without relying on predefined windows or fixed receptive fields. The structure of the DAT network is illustrated in Figure 6.

Dynamic Offset Learning and Semantic-Driven Feature Fusion significantly enhance detection accuracy in complex scenarios. As illustrated in Figure 6a, the module employs uniformly distributed reference points as a foundation and generates deformation vectors via a lightweight offset network (refer to Figure 6b). This network drives the sampling points to autonomously cluster in regions that are sensitive to pathological features. The offset network is designed using depthwise separable convolutions and nonlinear activation layers, where input features are hierarchically compressed to produce a high-precision offset field, thereby balancing computational efficiency with deformation sensitivity. The deformation points are central to multi-scale sampling, extracting lesion contour features through bilinear interpolation while fusing relative position encoding to construct spatially aware key/value pairs. The multi-head attention mechanism integrates deformation features and positional deviations, yielding a response matrix that is highly correlated with pathological characteristics. A dense reference point strategy is adopted to comprehensively cover the microscopic topology of lesions, autonomously suppressing non-pathological interference in occluded scenarios and enhancing semantic focus on visible regions.

#### 2.2.5. Asymptotic Feature Pyramid Network

In natural orchard environments, a significant challenge in citrus disease detection is the high false-detection rate associated with tiny lesions. Traditional detection methods often overlook these lesions due to insufficient feature information. When lesions are located at the edges of fruits or obscured by branches and leaves, existing approaches struggle to accurately identify them. The Asymptotic Feature Pyramid Network (AFPN) constructs a dynamic perception feature fusion system that adaptively adjusts feature extraction strategies based on the scale and location of lesions. By employing a learnable channel attention module, the network dynamically adjusts the fusion weights of features across different levels, enabling it to autonomously determine when to rely on high-resolution shallow features to capture the details of tiny lesions. Through an improved cross-scale connection method, AFPN facilitates more direct information interaction between the levels of the feature pyramid, thereby avoiding the information attenuation problem commonly encountered in traditional methods. Additionally, AFPN introduces a spatial attention mechanism in the detection Head, which allows the network to concentrate on potential lesion areas while effectively suppressing interference from complex backgrounds.

**Figure 7:**
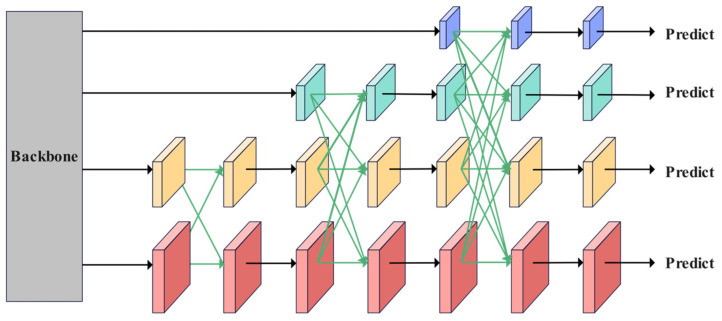
AFPN structure diagram.

The AFPN progressively extracts features through four consecutive downsampling stages (Stage 1 to Stage 4) based on the Backbone network. Each stage outputs its final layer of features, forming a multi-scale feature set {C2, C3, C4, C5}. A multi-scale pyramid is constructed using a phased progressive fusion mechanism, as illustrated in Figure 7. The low-level features (C2, C3) contain rich spatial detail, while the high-level features (C4, C5) encode more abstract semantic content. The network horizontally connects low-level features (C2, C3), aligns channels, and preserves detailed information to generate preliminary fusion results. Subsequently, this result interacts cross-level with deep features (C4, C5), dynamically unifying resolution through upsampling and downsampling, and gradually integrates semantics of different abstraction levels using skip connections. Finally, the top-level feature (C5) of the Backbone network is embedded into the pyramid structure, outputting {P_2_, P_3_, P_4_, P_5_} as input to the detection Head [41]. The architecture adopts a step-by-step fusion strategy that progresses from local to global, prioritizing the bridging of semantic gaps between adjacent layers before gradually extending to non-adjacent layers. This approach effectively mitigates feature conflicts caused by direct cross-layer fusion in traditional methods, demonstrating enhanced robustness, particularly in small target localization and dense scenes.

**Figure 8:**
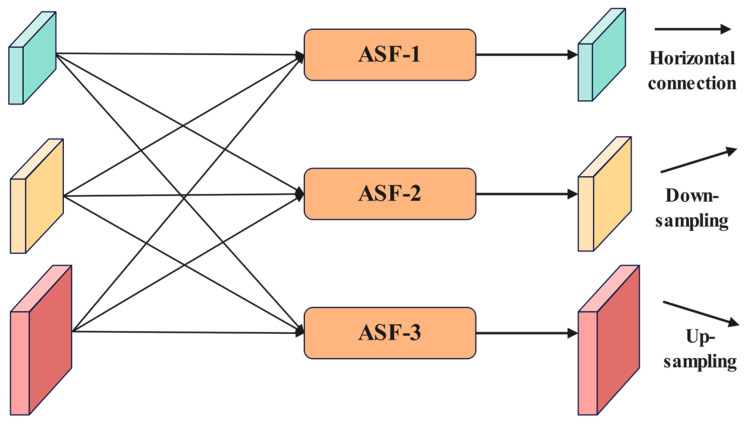
Adaptive space fusion operation.

As illustrated in Figure 7, the cross-level feature fusion of the Adaptive Feature Pyramid Network (AFPN) is achieved through Adaptive Spatial Feature Fusion (ASFF), indicated by the green arrows. The operational process of ASFF is further detailed in Figure 8. The traditional element-wise addition method for fusing multi-level features can inadvertently amplify noise due to conflicting information from different targets at the same spatial location. In contrast, ASFF introduces a dynamic weight allocation mechanism in the spatial dimension. For each level of features to be fused, a corresponding spatial attention map is generated through a lightweight convolutional layer, which is then normalized into weight coefficients using the Softmax function. As illustrated in Figure 8, within the three-level cascaded fusion framework, we denote $x_{ij}^{n\to l}$ as the feature vector transferred from hierarchy n to hierarchy l at spatial position (i,j) The resultant fused feature representation $y_{ij}^{1\to l}$ is derived through our proposed cross-layer aggregation strategy defined in Equation (3).

(3) $$y_{ij}^{l}=\alpha_{ij}^{l}\cdot x_{ij}^{1\to l}+\beta_{ij}^{l}\cdot x_{ij}^{2\to l}+\gamma_{ij}^{l}\cdot x_{ij}^{3\to l},$$

In Equation (3), the learnable parameters $\alpha_{ij}^{l}$, $\beta_{ij}^{l}$ and $\gamma_{ij}^{l}$ represent the multi-scale spatial attention coefficients corresponding to distinct hierarchical levels, where their inter-layer relationships are constrained by the normalization condition prescribed in Equation (4).

(4) $$\alpha_{ij}^{l}+\beta_{ij}^{l}+\gamma_{ij}^{l}=1,$$

This mechanism achieves cross-level semantic alignment through dynamic weight adjustment, effectively suppressing low-level noise in large target regions that are dominated by high-level features, while enhancing detail expression at small target edges that are sensitive to low-level features. Coupled with the fundamental feature transformation represented by the black arrows (1 × 1 convolution) in Figure 7, the AFPN only requires adjustments to the number of input features to adapt to pyramid structures at different levels. This ensures a balance between accuracy and robustness in multi-scale object detection under complex scenarios.

#### 2.2.6. Slide Loss Function

In object detection tasks, IoU [42,43] serves as a fundamental evaluation metric that measures localization accuracy by calculating the ratio of the intersection area to the union area of predicted and ground truth bounding boxes. However, its primary limitations include zero gradient responses for non-overlapping boxes and sensitivity to minor boundary shifts. To address these issues, Generalized IoU (GIoU) [44] introduces a minimum bounding box constraint, thereby expanding the optimization space by penalizing non-overlapping void regions between predicted and ground truth boxes. Building upon this, Distance IoU (DIoU) [45] incorporates a geometric distance constraint, integrating the Euclidean distance between the centers of bounding boxes into the loss calculation, which effectively enhances convergence efficiency. Complete IoU (CIoU) [46,47] further synthesizes three elements, overlap area, center distance, and aspect ratio alignment, constructing a multi-dimensional geometric alignment objective. Nevertheless, these methods still encounter the challenge of imbalanced regression difficulty in complex scenarios, such as multi-scale target coexistence or partial occlusion. In these situations, small targets are particularly susceptible to background noise interference, leading to localization jitter, while large targets are prone to local overfitting due to gradient dominance. The Slide Loss [48] introduces a dynamic gradient adjustment strategy that adaptively modifies the curvature of the loss function according to the scale characteristics of the target. This approach enhances the gradient contribution weight in regions with small targets while diminishing redundant optimization signals in larger targets. By employing an asymmetric weight distribution, it facilitates scale-aware bounding box regression. This design exhibits enhanced stability and generalization capabilities in challenging scenarios, such as dense occlusion and extreme scale distribution, positioning it as a contemporary solution for loss functions that effectively balances high precision with robust performance.

**Figure 9:**
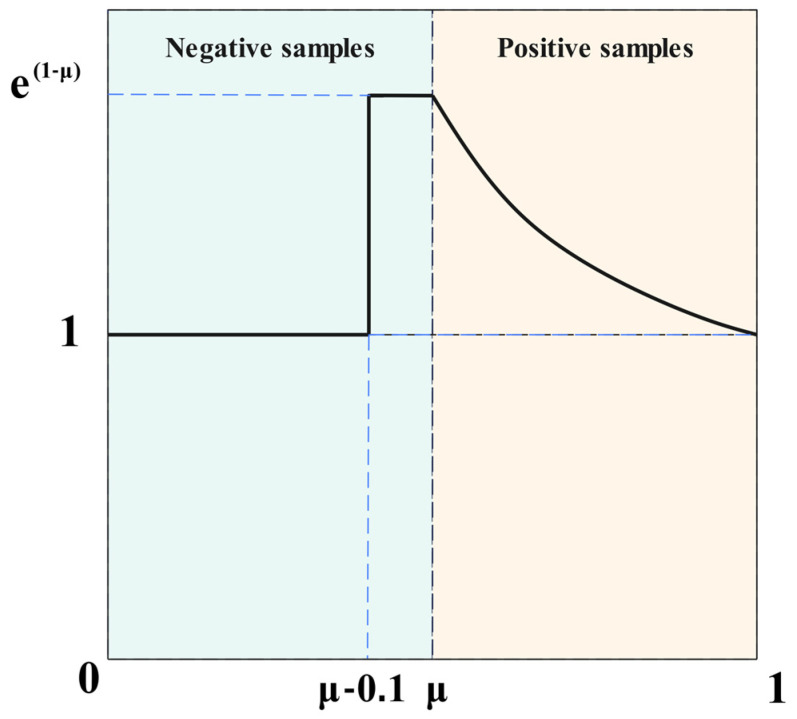
Schematic diagram of the Slide Loss function.

In this study, we adopt Slide Loss as the loss function, effectively transforming the object detection problem into a regression problem. Model optimization is achieved by minimizing the IoU between the predicted bounding box and the ground truth bounding box, as illustrated in Figure 9.

Slide Loss proposes a data-driven sample division mechanism that dynamically distinguishes low-confidence samples from high-confidence samples based on the IoU distribution between predicted boxes and ground truth boxes. Unlike traditional methods that rely on fixed threshold settings, this approach utilizes the mean IoU (*μ*) of all bounding boxes within the current batch as a dynamic boundary. It classifies ambiguous samples with IoU < *μ* as negative samples for optimization, while samples with IoU ≥ *μ* serve as positive supervision signals. This adaptive threshold generation mechanism effectively mitigates the model’s sensitivity and scene generalization bottlenecks caused by manually set confidence thresholds, particularly in scenarios with dense small objects or severe occlusions. By dynamically adjusting the ratio of positive to negative samples, it suppresses the gradient dominance phenomenon associated with simple samples. Slide Loss constructs a nonlinear weight mapping function and implements a gradient rescaling strategy for transition region samples near dynamic thresholds. This enhancement bolsters the model’s feature recognition capability for boundary-ambiguous samples during training while mitigating the risk of overfitting to extremely challenging samples. This design achieves balanced optimization of multi-scale objectives through end-to-end gradient modulation, significantly improving localization stability and generalization capability in complex scenarios while maintaining a high recall rate for the detector.

## 3. Results

### 3.1. Experimental Platform

The model experiments conducted in this study were performed on the Windows 11 operating system, utilizing the following hardware specifications: an AMD Ryzen 9 7945HX processor, an NVIDIA GeForce RTX 4060 graphics card, and 32 GB of RAM. The programming language employed was Python 3.10.10, while the deep learning framework utilized was PyTorch 1.13.1. The development environment was PyCharm (2021.2.2), and the Compute Unified Device Architecture (CUDA) version was 12.0. The batch size is set to 4, the learning rate is set to 0.01, and Stochastic Gradient Descent (SGD) is employed as the optimizer. The number of training iterations is fixed at 500.

### 3.2. Model Evaluation Metrics

This experiment utilizes precision (P), recall (R), average precision (AP), and mean average precision (mAP) to assess the model’s performance. Notably, mAP@50 demonstrates stability in conventional scenarios under a lenient localization criterion with an IoU threshold of 0.5. Conversely, mAP@50:95 computes the mean localization accuracy across various stringent levels by applying progressive IoU thresholds ranging from 0.5 to 0.95 in increments of 0.05, thereby evaluating the model’s adaptability to extreme scenarios. The specific calculations are presented in Equations (5)–(8).

(5) $$P=\frac{T_{P}}{T_{P}+F_{P}}$$

(6) $$R=\frac{T_{P}}{T_{P}+F_{N}}$$

(7) $$AP=\int_{0}^{1}P\left(R\right)dR$$

(8) $$mAP=\frac{\sum_{i=1}^{C}AP\left(i\right)}{C}$$

### 3.3. Training Results

**Figure 10:**
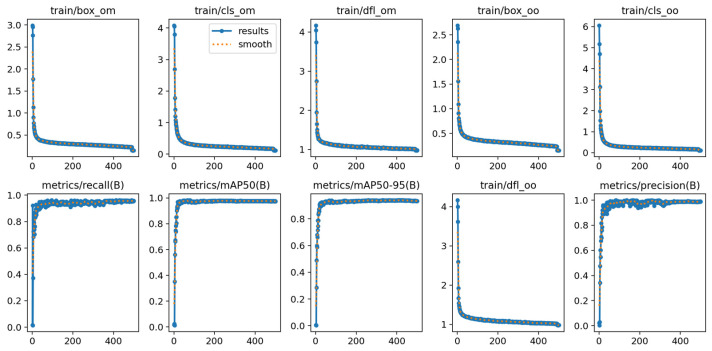
Loss function result graphs.

After 500 rounds of model training iterations, the YOLOv10-LGDA model exhibits exceptional performance, achieving an accuracy of 98.7%, a recall rate of 95.9%, an mAP@50 of 97.7%, and an mAP@50:95 of 94%. This performance enhancement is closely associated with the dynamic evolution of the loss function curve. The loss function curve visually represents the dynamic characteristics of the model optimization process and plays a crucial role in guiding training and optimization [49]. The effective reduction in loss values signifies a high degree of fit between the predicted results and the true distribution, thereby reflecting the model’s precise modeling capability for the training samples. In this experiment, the loss value consistently and steadily decreased throughout the training process, with its convergence trajectory intuitively illustrating the model’s adeptness at modeling the distribution characteristics of the training samples. The gradient propagation mechanism continuously drove the network parameters to optimize iteratively towards a more generalizable direction, ultimately achieving a high degree of fit between the predicted results and the true labels. The loss function is depicted in Figure 10.

### 3.4. Model Performance Analysis and Ablation Study

Improvements were made to the YOLOv10 model, and the results of each enhancement were analyzed. The data outcomes are presented in Table 1.

Table 1 presents the experimental results of the YOLOv10 model enhanced with LDConv, GFPN, DAT, and AFPN modules. The checkmark (√) indicates the inclusion of a module, while the cross (×) denotes its exclusion. The ablation study demonstrates that the enhancements made to the modules effectively improve the detection performance of the YOLOv10 model. The gradient optimization and cross-level fusion design strengthen the complementarity of multi-scale features, resulting in superior performance in key metrics such as accuracy, recall, and average precision. Analyzing the experimental data in Table 2 reveals that after incorporating LDConv, the recall rate increased by 0.4% and the mean average precision at IoU 0.50 (mAP@50) improved by 0.7%. Following the integration of LDConv and GFPN, the accuracy rate rose by 2.6%, and mAP@50 increased by 2.7%. Moreover, the fusion of GFPN, DAT, and AFPN led to an overall enhancement in model performance, with accuracy, recall, mAP@50, and mAP@50:95 increasing by 3.8%, 2.8%, 4.1%, and 1.8%, respectively. Finally, building upon the enhancements from GFPN, DAT, and AFPN, the addition of the LDConv module further optimized the previous improvements, resulting in an additional increase of 0.6% in average precision (mAP@50:95), thereby significantly enhancing the model’s capability to detect citrus disease images.

**Figure 11:**
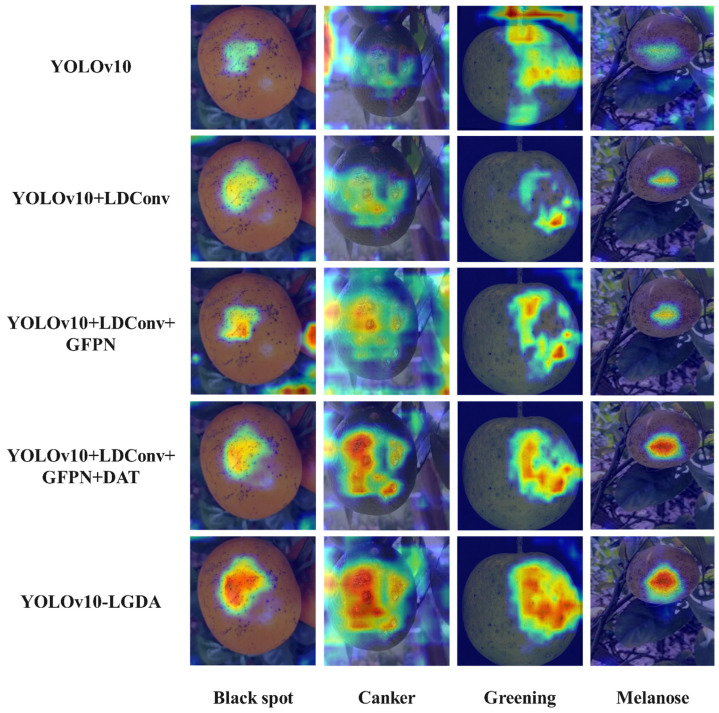
Comparison of heatmaps from different improved models.

To validate the perception and localization capabilities of the YOLOv10-LGDA model concerning citrus fruit disease features, this paper employs the Grad-CAM heatmap analysis method to assess the effectiveness of various module combinations. Unlike traditional detection tasks that depend on manual expertise for feature analysis, Grad-CAM generates a heat distribution map that covers the fruit surface by backpropagating gradient information and globally averaging the weights of feature maps. In the comparative experiments, all heatmaps were extracted from the final output layer, specifically the C5 layer, of the Backbone network. This heatmap utilizes a color gradient transitioning from cool to warm tones to visually quantify the model’s attentional intensity towards different pathological regions. Samples of four diseases—citrus black spot, citrus canker, citrus greening, and citrus melanose—are selected as the subjects of this research, with the results presented in Figure 11. The output heatmap of the YOLOv10 network (A) is characterized by a scattered distribution dominated by cool tones, indicating insufficient focus on diseases and a tendency to misdetect similar background regions. In contrast, the output heatmap of YOLOv10 + LDConv (B) demonstrates a shift towards the warm color spectrum, beginning to concentrate on small-sized lesions; however, the core disease regions remain predominantly yellow/green. The output heatmap of YOLOv10 + LDConv + GFPN (C) shows improved alignment with the contours of lesions, while the output heatmap of YOLOv10 + LDConv + GFPN + DAT (D) exhibits enhanced red saturation in the disease feature regions, signifying a concentrated attention on citrus disease features and a noticeable suppression of background interference. Finally, the output heatmap of YOLOv10 + LDConv + GFPN + DAT + AFPN (E) reveals a significant pathological focus, with markedly improved precision in lesion boundary locking, achieving a high degree of localization accuracy with pathological features.

**Figure 12:**
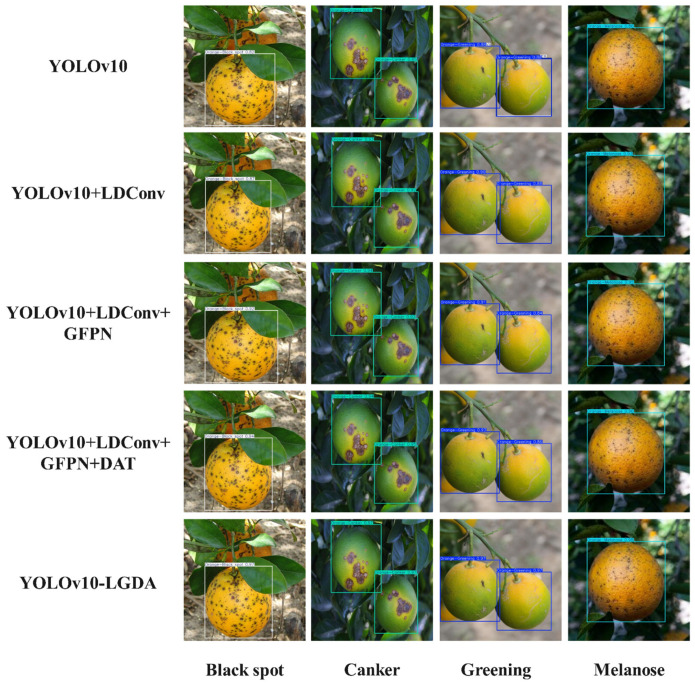
Comparison of effects before and after YOLOv10 improvement.

To further validate the detection performance of YOLOv10-LGDA in identifying citrus fruit diseases across diverse background environments, densely diseased images were selected from the dataset for testing, and their respective confidence levels were recorded. The baseline model, YOLOv10, demonstrates robust performance with samples exhibiting simple backgrounds and prominent disease spots, achieving detection confidence levels exceeding 0.8 for four typical diseases. However, it reveals limited generalization capabilities when confronted with complex pathological features, as evidenced by fluctuating confidence levels and decreased recognition rates in the presence of small disease spots, densely clustered lesions, or interference from background similarities. In contrast, the improved YOLOv10-LGDA model achieves higher confidence scores in detecting images of citrus fruit diseases, reaching a peak detection confidence of 0.98, thereby significantly enhancing the performance of citrus disease image recognition. Specific results are illustrated in Figure 12.

### 3.5. Comparative Experiment on Performance of Different Network Models

To evaluate the recognition capability of the improved YOLOv10-LGDA model for citrus fruit diseases, seven network models were selected for comparative experiments: Faster R-CNN, SSD, YOLOv5, YOLOv7, YOLOv8, YOLOv10, and YOLOv10-LGDA. All models were tested in the same training environment and with the same dataset. Performance evaluation metrics included accuracy, recall, mAP@50, and mAP@50:95. The results of the comparative experiments are presented in Table 2. As illustrated in Table 2, Faster R-CNN and SSD demonstrate relatively poor detection performance, with the highest average detection accuracy only reaching approximately 85%. In contrast, the YOLO models exhibit superior detection performance, with YOLOv10-LGDA achieving the best results. Its mAP@50:95 surpasses that of Faster R-CNN, SSD, YOLOv5, YOLOv7, and YOLOv8 by 10.4%, 9.9%, 5.4%, 4.7%, and 2.6%, respectively.

**Figure 13:**
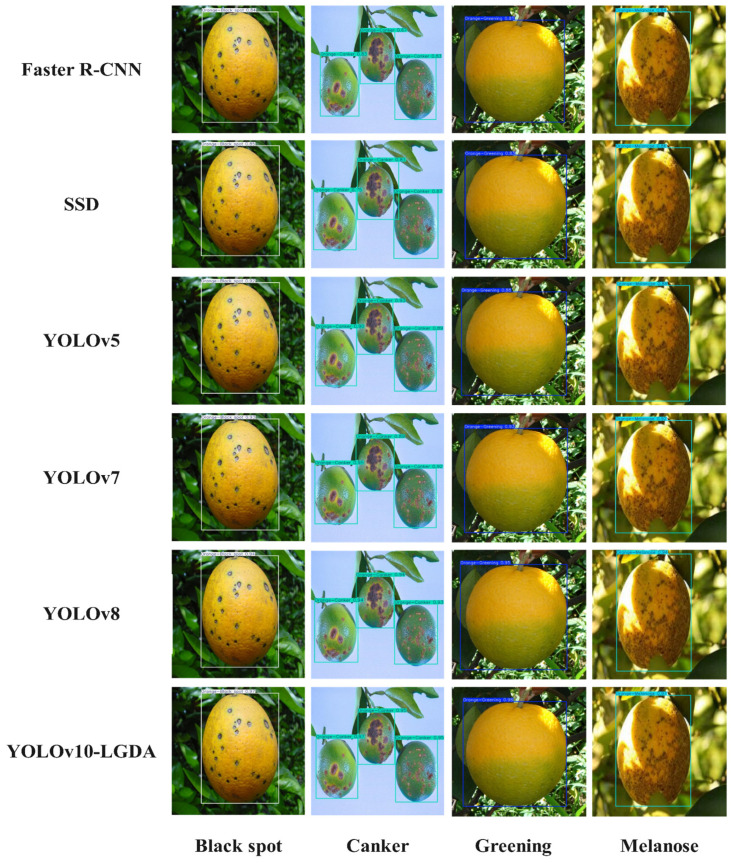
Comparison of the effects of different network models.

To further validate the detection performance of the improved YOLOv10-LGDA model for various citrus diseases, we will observe its confidence levels in comparison with the previously mentioned models. The results indicate that, for the four selected citrus diseases under investigation, the confidence levels of the Faster R-CNN and SSD models were generally lower than those of the YOLO series models. Notably, the improved YOLOv10-LGDA achieved the highest recognition rate, with confidence levels consistently reaching 0.96. The specific results are presented in Figure 13.

In summary, the enhanced YOLOv10-LGDA model significantly improves the recognition accuracy of disease spots and moldy areas in citrus disease detection by strengthening multi-scale feature fusion and anti-interference capabilities. Even in complex scenarios characterized by uneven lighting or overlapping branches and leaves, it can reliably distinguish subtle differences between similar diseases. The optimized network structure effectively reduces the misjudgment rate of healthy areas, achieving higher precision, recall, and average precision, with detection performance that surpasses other object detection models.

## 4. Discussion

The improved YOLOv10 model proposed in this study systematically enhances the accuracy and scene adaptability of citrus disease detection through multi-module collaborative optimization. Experimental results demonstrate a significant increase in the detection accuracy of the improved YOLOv10-LGDA model compared to the original YOLOv10 model. Furthermore, when juxtaposed with mainstream object detection models such as Faster R-CNN, SSD, YOLOv5, YOLOv7, and YOLOv8, its innovative architecture exhibits notable advantages in agricultural scenarios. This model achieves dynamic adaptation across the entire process, from feature extraction to target analysis, thereby providing a novel architectural paradigm for complex object detection in agricultural settings.

The current study presents several limitations. Firstly, the dataset encompasses images of only four disease types, black spot, canker, greening, and melanose, leaving the detection capabilities for other prevalent citrus diseases unverified. Secondly, the image data lacks adequate representation of certain disease morphologies, and there is an insufficient number of dynamic interference images from real orchards, such as those with multi-angle lighting and overlapping fruit lesions. This deficiency restricts the model’s generalization in practical applications. Although the model has demonstrated efficient performance on GPU platforms, its real-time performance on edge devices necessitates further optimization.

Future research will focus on synthesizing diverse lesion data using Generative Adversarial Networks, reconstructing models for lightweight deployment, and collaborating with agricultural institutions to create datasets that encompass multiple diseases and growth stages. This approach aims to expand the scope of multi-disease detection and optimize edge deployment, thereby enhancing environmental generalization capabilities. Particular emphasis will be placed on field applications, ensuring that edge devices balance speed and accuracy to address challenges such as lighting variations, network connectivity, and limited computational resources. These advancements will significantly facilitate the transition of agricultural visual inspection technologies from laboratory settings to large-scale field applications, thereby promoting the widespread adoption of smart agriculture.

## 5. Conclusions

This paper addresses the issue of citrus fruit disease detection by proposing an improved YOLOv10-based disease detection algorithm. The algorithm replaces the Backbone convolutional layer with LDConv and introduces GFPN in the Neck to enhance the feature fusion structure. Additionally, the DAT mechanism is incorporated to enable the model to adaptively focus on key areas affected by citrus diseases. The AFPN is utilized to improve the detection Head, thereby strengthening the complementary semantic and positional information of features at different levels. This approach effectively resolves the issues of low recognition rates in traditional algorithms for citrus fruit disease detection, as well as the problem of false detections in small target detection tasks.

The improved model, YOLOv10-LGDA, achieved notable performance metrics, including accuracy, recall, mAP@50, and mAP@50:95 levels of 98.7%, 95.9%, 97.7%, and 94.0%, respectively. These results indicate an increase of 4.2%, 3.8%, 4.5%, and 2.4% compared to the original YOLOv10 model. Through collaborative optimization of the modules, the detection performance has been significantly enhanced, offering an efficient solution for real-time agricultural detection tasks.

## Figures and Tables

**Table 1:** Ablation experiment.

LDConv	GFPN	DAT	AFPN	P/%	R/%	mAP@50/%	mAP@50:95/%
×	×	×	×	94.5	92.1	93.2	91.6
√	×	×	×	95.4	92.5	93.9	91.9
×	√	×	×	95.9	92.8	94.2	92.1
×	×	√	×	94.8	92.6	93.5	91.8
√	√	×	×	97.1	93.6	95.9	92.7
×	√	√	×	96.9	93.9	95.8	92.4
×	×	√	√	97.6	94.1	96.2	92.6
×	√	√	√	98.3	94.9	97.3	93.4
√	√	√	√	98.7	95.9	97.7	94.0

Note: √, uses this module; ×, does not use this module.

**Table 2:** Comparison results of different network models for citrus disease detection.

Model	P/%	R/%	mAP@50/%	mAP@50:95/%
Faster R-CNN	84.2	81.7	84.1	83.6
SSD	82.1	85.1	85.3	84.1
YOLOv5	88.5	87.5	89.5	88.6
YOLOv7	90.1	89.3	90.7	89.3
YOLOv8	93.6	91.3	92.2	91.4
YOLOv10	94.5	92.1	93.2	91.6
YOLOv10-LGDA	98.7	95.9	97.7	94.0

## Data Availability

The data provided in this study can be obtained from the corresponding author upon request.
